# Ancient dog diets on the Pacific Northwest Coast: zooarchaeological and stable isotope modelling evidence from Tseshaht territory and beyond

**DOI:** 10.1038/s41598-020-71574-x

**Published:** 2020-10-01

**Authors:** Dylan Hillis, Iain McKechnie, Eric Guiry, Denis E. St. Claire, Chris T. Darimont

**Affiliations:** 1 Department of Anthropology, University of Victoria, 3800 Finnerty Rd, Victoria, BC V8P 5C2 Canada; 2 Department of Geography, University of Victoria, Victoria, BC Canada; 3 Hakai Institute, Heriot Bay, Quadra Island, BC Canada; 4 Department of Anthropology, Trent University, Peterborough, ON Canada; 5 School of Archaeology and Ancient History, University of Leicester, Leicester, UK; 6 Department of Anthropology, University of British Columbia, Vancouver, BC Canada; 7 Tseshaht First Nation, Port Alberni, BC Canada; 8 Coast Heritage Consulting, Victoria, BC Canada; 9 Raincoast Conservation Foundation, Sidney, BC Canada

**Keywords:** Environmental economics, Geochemistry, Macroecology, Palaeoecology, Stable isotope analysis, Ecology, Ocean sciences

## Abstract

Domestic dogs are frequently encountered in Indigenous archaeological sites on the Northwest Coast of North America. Although dogs depended on human communities for care and provisioning, archaeologists lack information about the specific foods dogs consumed. Previous research has used stable isotope analysis of dog diets for insight into human subsistence (‘canine surrogacy’ model) and identified considerable use of marine resources. Here, we use zooarchaeological data to develop and apply a Bayesian mixing model (MixSIAR) to estimate dietary composition from 14 domestic dogs and 13 potential prey taxa from four archaeological sites (2,900–300 BP) in Tseshaht First Nation territory on western Vancouver Island, British Columbia, Canada. Two candidate models that best match zooarchaeological data indicate dogs predominantly consumed salmon and forage fish (35–65%), followed by nearshore fish (4–40%), and marine mammals (2–30%). We compared these isotopic data to dogs across the Northwest Coast, which indicated a pronounced marine diet for Tseshaht dogs and, presumably, their human providers. These results are broadly consistent with the canine surrogacy model as well as help illuminate human participation in pre-industrial marine food webs and the long-term role of fisheries in Indigenous economies and lifeways.

## Introduction

Although Indigenous communities on the Northwest Coast of North America have been classically described as ‘hunter-gatherers’ or ‘fisher-hunter-gatherers’, they maintained disproportionately high population densities, extensive trade networks, and elaborate territorial and governance structures with a corresponding influence on coastal landscapes^1^. A less well recognized component of cultural practices in this region is the care and maintenance of domestic dog (*Canis familiaris*) populations. Domestic dogs occur frequently in the early historic and oral historical accounts of the social and economic practices of Indigenous communities on the coast^2,3^. The skeletal remains of dogs, including purposeful interments, are regularly encountered in archaeological site deposits dating back to the earliest sites with preserved fauna^3,4^. Given that dogs depended on human communities for provisioning and protection from predators such as wolves, it has been postulated that the diet of dogs can reflect human subsistence practices through provisioning as well as scavenging of human refuse^5^. One method by which researchers have generated insight into this relationship has been stable isotope analysis^6,7^.

Stable isotope analysis (SIA) estimates the relative contribution of foods that comprise an organism’s diet and is a widely used method in ecology, archaeology, paleobiology, and forensics. Isotopic signatures can estimate diets among populations of the past via use of bone collagen and other preserved tissue of consumers and their foods. Two of the most commonly analyzed isotopes include carbon (*δ*^13^C), which can be used to assess whether an animal’s protein is derived from marine or terrestrial sources^8^, and nitrogen (*δ*^15^N) isotopes, which reflect trophic position^9^ as well as extent of terrestrial vs marine consumption^10^. Given that SIA of human diet involves the destructive analysis of ancestral human remains, such research may not align with the interests of Indigenous peoples. In light of this, SIA has been used on domestic dogs to infer human subsistence activities and for inferences into the role of humans in pre-industrial food webs^5,11,12^.

Previous work has applied SIA on dog remains to infer dog and human diets at specific sites and time periods. A pathbreaking study on British Columbia’s (BC’s) central coast used stable carbon and nitrogen isotopes to evaluate the dietary composition of domestic dogs at Namu. There, isotopic data from the skeletal remains of both human^8^ and dog^7^ populations revealed highly marine-derived diets and evidence of a sustained maritime subsistence economy over the past six millennia. Isotopic analysis of dog bones from a site on northern Galiano Island in the Salish Sea (BC) additionally highlighted the role of marine protein in human subsistence and domestication practices, which was provisionally linked to the consumption of herring and salmon, common organisms in the zooarchaeological record^13^. Along the Lower Columbia River in Washington State, Ames et al.^14^ showed that dog stable isotope compositions suggested a balance between marine and terrestrial food use. These findings were interpreted alongside zooarchaeological data as linked to salmon, smelt, and sturgeon fisheries. In the interior plateau region of the Fraser River, studies of dog diets revealed distinctly marine signatures consistent with human stable isotopic compositions and access to migratory salmon^15,16^. These multiple research efforts have established that both humans and dogs predominantly depended on marine dietary protein, even in areas hundreds of kilometers away from the coast^17,18^.

Collectively, these case studies offer detailed perspective on resource use at specific sites, yet new approaches are required to increase resolution of our understanding and to examine potential differences among sites and over time. Such analyses can refine our understanding of spatial and temporal variation in use of food resources by Indigenous societies on the Northwest Coast. Consequently, regional compilations of archaeological samples across space and time, and new methods that quantitatively estimate the relative contributions of candidate foods, provide more detailed inference than graphical comparisons between stable isotopic compositions and prey data. Limitations of prior studies often include a lack of sufficiently detailed and regionally comparable isotopic baseline data necessary for assessing dietary variability (although see^19^), as well as relatively small collections of dog remains. Investigations to date have also focussed on individual sites and have not employed quantitative methods to compare isotopic data from dog remains and potential food taxa to infer diet. In contrast, Bayesian ‘mixing models’, can estimate the relative contribution of pre-determined food groups to a consumer’s diet^20^ at multiple sites and time periods provided sufficient baseline data.

Leveraging published data and insight from past work and applying new mixing model methods, we combine stable isotope analysis of dog bone collagen with a suite of prey isotopic compositions. We estimated the diets of dogs using a Bayesian mixing model informed by zooarchaeological evidence, to improve detail into human participation in coastal food webs and variability across divergent geographies and past ecological conditions. Accordingly, in this study, we use stable isotope compositions from a variety of terrestrial and marine taxa to interpret dog diets from a previously unexamined region of the Northwest Coast. Utilizing site specific zooarchaeological data, we compare model estimates to this independent data source to identify preferred models. These data provide new insight into variation in coastal food webs and human resource use across western North America, as well as identify spatially- and temporally-detailed information about dog and human resource use in the Broken Group Islands, Tseshaht Territory on western Vancouver Island, BC.

## Materials and methods

### Sample description

**Figure 1:**
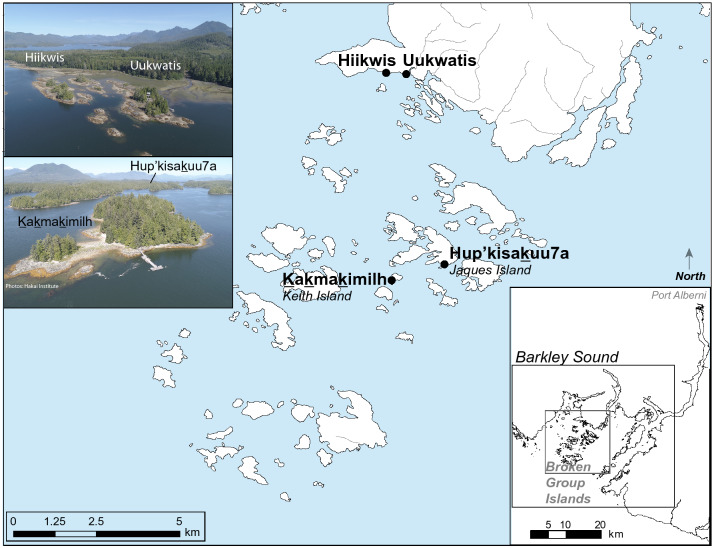
Map of the Broken Group Island study area in Tseshaht Territory on western Vancouver Island, British Columbia, Canada. Inset image credit: Hakai Geospatial.

We measured the isotopic composition of 14 zooarchaeologically identified domestic dogs (*Canis familiaris*) from four late-Holocene settlement sites in Tseshaht First Nation territory in Barkley Sound, BC. Two sites are located on small islands (< 5 km^2^) within the Broken Group, while two others are situated < 1 km apart on adjacent Vancouver Island (Fig. 1). Three of the four ethnographically described settlement sites are roughly contemporaneous (1,200–300 cal BP), while specimens from a deposit at one site (Uukwatis) date to an earlier period (2,880–1,830 cal BP; see Supplementary Table S1). Archaeological deposits at these locations are consistent with ethnographic descriptions of multi-family villages which regularly had dogs^21^. The remains of a single dog interment, recovered from Kakmakimilh (Keith Island, 306T), has been morphologically and osteometrically identified as a ‘wool’ dog^3^ with an estimated body mass of 16.1 kg^22^.

We additionally analyzed 141 samples of potential foods from zooarchaeologically-identified vertebrate taxa, representing 8 fish and 4 mammal genera, which are well represented within archaeofaunal assemblages in the Broken Group Island study area. All specimens were obtained from archaeological sites in the same study area and date between approximately 2,800 and 250 cal BP^23–25^. Samples were selected based on minimum number of individuals estimated per archaeological context to minimize the probability that duplicate results might be obtained from a single animal. Sampling of faunal remains was more intensive for commonly occurring genera with numerous species (i.e., *Oncorhynchus* spp*.*, *Sebastes* spp*.*), with the intention to estimate isotopic variation within individual taxa more precisely. Whereas it is possible that some of the isotopic variation in this dataset could reflect large-scale chronological differences, these data provide a late-Holocene perspective on isotopic variation in candidate foods for analyzing dog diets within the study area.

### Sample preparation

Preparation for isotopic analysis followed standard bone collagen extraction and calibration methods^26–28^. Collagen extractions were conducted in multiple stages at the University of British Columbia’s Laboratory of Archaeology (UBC LOA) stable isotope facility, housed in the Museum of Anthropology, Vancouver, Canada. Bone specimens were cleaned of surface residue by brushing and washing with tap water, rinsed with deionized water, and air dried. Subsamples of 50–100 mg were taken from dried specimens for collagen extraction. To account for potential lipid contamination in fish bones, fish samples were placed in an ultrasonic bath and soaked in 5–10 ml 2:1 chloroform–methanol for 15–45 min (solution refreshed every 15 min until the solution remained clear)^27^. Bone samples were demineralized in 0.5 M Hydrochloric acid (HCl) at 4 °C for ~ 12–15 h. Samples were then rinsed with Mili-Q water to neutrality. Humic acids were removed by soaking the samples in 0.1 M Sodium hydroxide (NaOH) at room temperature in an ultrasonic bath for successive ~ 10–15 min periods (until the solution remained clear). Samples were then rinsed to neutrality with Milli-Q water. Sample residue was solubilized in 10^−3^ M HCl (pH 3), heated to 75 °C for 48 h. Gelatinized collagen was then freeze dried (lyophilized); lyophilized collagen was then weighted into 0.5 mg samples and placed into tin capsules for isotopic and elemental analysis.

### Stable isotope analysis

For *δ*^13^C and *δ*^15^N analyses, 149 baseline faunal samples as well as one dog were measured using an elementar vario MICRO cube elemental analyzer coupled to IsoPrime isotope ratio mass spectrometer in continuous flow mode (UBC LOA). Elemental and isotopic compositions for 17 dogs and 6 in-house (UBC LOA) collagen standards were analyzed with a Costech ECS4010 elemental analyzer coupled to a Thermo Scientific Delta V mass spectrometer with ConFlo IV interface, at the Department of Soil Sciences, University of Saskatchewan (USask). Elemental and isotopic compositions for 5 source samples and 30 in-house (UBC LOA) collagen standards were analyzed with a PDZ Europa ANCA-GSL elemental analyzer interfaced to a PDZ Europa 20–20 isotope ratio mass spectrometer, at the Department of Plant Sciences stable isotope facility, University of California Davis (UC Davis). Stable carbon and nitrogen isotope compositions were calibrated relative to Vienna Pee Dee Belemnite (VPDB) and atmospheric nitrogen (AIR), respectively (see Supplementary Information S1 for more on measurement accuracy and precision). Of 154 samples analyzed, 13 potential food samples and 4 putative dogs were excluded from further analysis due to C:N_Atomic_ ratios that fell outside the established range for well-preserved bone collagen^29^ or misidentification of often fragmentary osteological specimens. For all other samples, collagen quality control indicators suggested excellent preservation, producing acceptable C:N_Atomic_, %C, and %N values (see Supplementary Information S1).

### Stable isotope mixing model

To consider potential dietary inputs, we aggregated candidate food groups, representing local taxa with shared ecological traits and habitat preferences. Archaeological samples from Broken Group Islands in Tseshaht territory (*n* = 141) were further supplemented with 166 regionally comparable stable isotopic compositions representing potential foods (see Supplementary Table S2)^30–32^. Using isotopic and ecological information, taxa with a high relative abundance in the region’s zooarchaeological record (8 fish, 4 mammal and 1 invertebrate genera) were aggregated into 5 isotopically distinct groups in order to generate the basis for modelling dietary contributions (see Supplementary Table S3). These food groups included marine mammal, nearshore fish, salmon/forage fish, shellfish, and terrestrial mammal.

Stable isotope compositions for dogs and their potential foods were analyzed in an open source Bayesian stable isotope mixing model (MixSIAR) that uses simulation to derive estimates of the relative contribution of defined food groups in a given consumer diet, while accounting for uncertainty in food source estimates^20^. The SIAR Markov chain Monte Carlo algorithm was run for 3,000,000 iterations per sample configuration. We used trophic discrimination factor (TDFs) to correct for differences in isotopic discrimination between the consumers (dog) and food sources. As a correction value is currently not available for domestic dogs, we drew upon a number of published studies that describe wolf-prey isotopic discrimination^9,33,34^. We used TDFs of − 1.0*‰* for *δ*^13^C; and − 3.6*‰* for *δ*^15^N. To estimate late-Holocene *δ*^13^C values for modern shellfish samples, we corrected for the Suess effect (+ 1.0*‰*) and a diet-collagen discrimination factor (+ 3.7*‰*); we assumed modern *δ*^15^N values for shellfish were applicable to the late-Holocene^35^. Shellfish tissue samples were not subject to acid or lipid extraction^36^. All statistical analysis was conducted with MixSIAR (v 3.1) in *R* (v 3.4.3).

**Figure 2:**
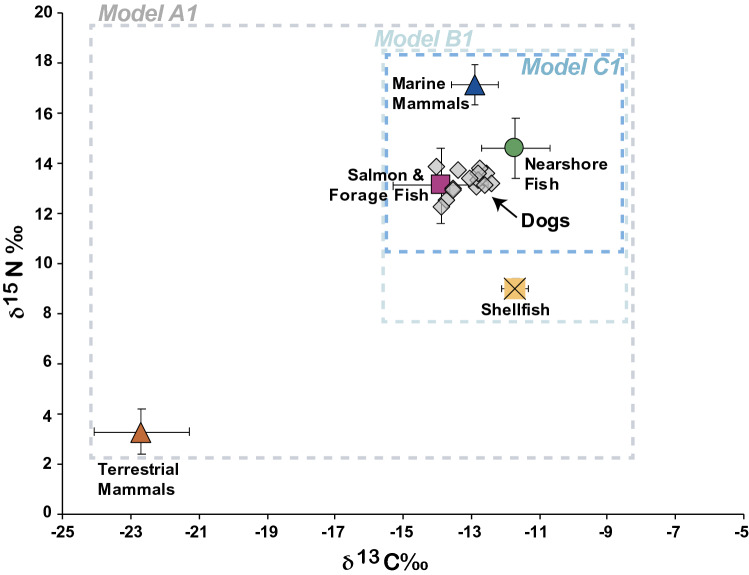
Isotopic distribution for 14 examined Broken Group Island dogs in comparison with regional archaeological isotope values. Model A1 includes terrestrial mammals, Model B1 focuses exclusively on the isospace of marine animals, while Model C1 excludes shellfish. We applied trophic discrimination factors of 1.0‰ for *δ*^13^C and 3.6‰ for *δ*^15^N to dog isotope values to correct for isotopic discrimination between consumers and food sources^9,31,32^.

To develop the MixSIAR model approach, we obtained stable isotope compositions from taxa that are present in the ethnographic and zooarchaeological record and represent site-specific human resource use. We focused isotopic sampling on taxa with the highest relative abundance and ubiquity in the study region^37,38^ as well as site-specific zooarchaeological assemblage data^23,39,40^, making the assumption that these food items were the most commonly available foods to ancient dog populations at these residential sites. Based on an examination of the distribution of isotopic values for potential foods and ecological habitat preferences, we identified a number of groupings that exhibited similar stable isotope compositions to develop isotopic prey categories for these groups (see Supplementary Table S4). We then developed three classes of models (A, B and C), which reflect nested scales of dietary profiles. These models used five isotopically distinct food groups in various configurations to derive food source estimates for three potential dietary scenarios (Fig. 2).

Across the suite of models, we identified outputs that exhibited probability distributions and provided comparatively narrow predictive ranges in *δ*-space (‘niche width’^41^). We then compared these models alongside isotope plot associations, identifying specific model forms that encompassed multiple scales of analytical resolution that could be evaluated against the zooarchaeological assemblage data. Owing to narrower ranges of isotopic compositions for potential food sources in the marine environment, the more refined class of B models excluded terrestrial mammals so as to distinguish marine food inputs. To further resolve the potential contributions of specific marine inputs, we removed shellfish from the C models and evaluated the effect this has on model output. For the 14 dogs, seven ‘populations’ were considered for each class of models (13 models in total); including all (a) Broken Group Islands (BGI; *n* = 14 dogs), specific archaeological sites (b) Kakmakimilh (Keith Island; *n* = 2 dogs), (c) Hup’kisakuu7a (Jaques Island; *n* = 1 dog), (d) Hiikwis (*n* = 2 dogs), (e) Uukwatis (*n* = 9 dogs), and over two time periods (f) period 1 (*n* = 9 dogs) and (g) period 2 (*n* = 5 dogs).

### Chronology and radiocarbon dating

Individual specimens used for stable isotope analysis originated from multiple late Holocene archaeological deposits in Barkley Sound, BC. Specimen ages are constrained by radiocarbon dating and geomorphological context, predominantly on stratigraphically associated terrestrial charcoal and/or terrestrial animal (deer) bone. Analyses were conducted at Lalonde AMS Lab at the University of Ottawa, Beta Analytic Inc. and the Keck Carbon Cycle AMS lab at UC Irvine. Radiocarbon results associated with the wool dog internment on Kakmakimilh date to approximately 670–550 calibrated yr BP (Supplementary Table S1). Other dog specimens were obtained from archaeological deposits that had charcoal dates from a stratigraphic sequence. Within the examined samples, the notable chronological distinction is between dog specimens from Uukwatis, which date to ca. 2,880–1,830 cal BP^24,39^, and all other samples, which date between approximately 1,200–300 cal BP (Supplementary Table S1).

## Results

### Regional variation

**Figure 3:**
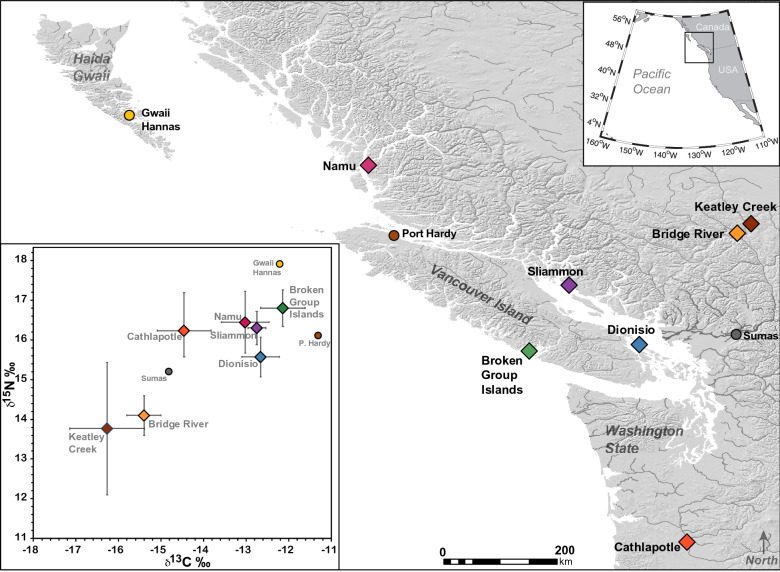
Bone collagen stable isotopic compositions from previously examined archaeological domestic dog samples on the Northwest Coast (Supplementary Table S7) in comparison with the Broken Group Islands, Tseshaht territory, dog population. Circles indicate single values while diamonds indicate multiple values with standard deviations.

The average and standard deviation in *δ*^13^C and *δ*^15^N across the dog populations were – 12.14 ± 0.52‰ and + 16.8 ± 0.46‰, respectively, reflecting a diet predominantly comprised of marine foods (Fig. 2). Compared to isotopic data from dogs sampled elsewhere on the Northwest Coast, the Broken Group Island dogs, in Tseshaht territory, had broadly elevated *δ*^13^C and *δ*^15^N values (Fig. 3). The distinct isotopic position of Broken Group Island dogs reflects an environment characterized by long food chains involving a mix of nearshore kelp-based and pelagic carbon^42,43^. As indicated in Fig. 3, comparison to mid-to-late Holocene dog populations from elsewhere on the Northwest Coast exhibited carbon and nitrogen isotopic compositions that are consistent with consumption of resources from a broad range of environments, including riverine, inner coastal, and exposed outer coast ecosystems^44^.

### Broken Group Island archaeological sites: summary across sites

**Figure 4:**
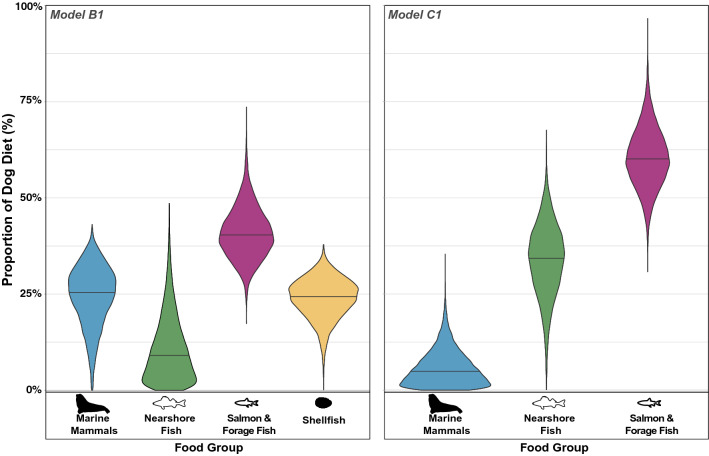
Violin plots representing dietary model estimates for Tseshaht dogs (n=14) as represented by model B1 (left) and model C1 (right) with horizontal lines indicating median (50%) probabilities (see Table 1).

Across candidate MixSIAR models in which multiple food groups were considered, we focus on median (50%) and interquartile range (25–75%) estimates of dietary consumption for models B1 and C1 (Table 1). Results for model B1 suggest that the consumption of each potential marine food group were broadly similar. Salmon/forage fish provided the largest source (40%, 36–45%) of diet, followed by marine mammals (26%, 20–30%) and shellfish (24%, 21–28%) (Table 1, Fig. 4). Nearshore fish (9%, 4–15%) and terrestrial mammals (model A1, 6%, 4–8%) comprised modest contributions. Results for model C1, which focused on the contribution of specific fish and marine mammals (Fig. 4), indicated that dog diets were predominantly comprised of salmon/forage fish (60%, 55–65%) followed more distantly by nearshore fish (34%, 28–40%) and marine mammals (5%, 2–8%). This latter model provided the tightest predictive ranges and is best supported by the similarity of stable isotope compositions to model predictions as well as zooarchaeological abundance data for these vertebrate taxa. However, because the C1 model excluded shellfish it may significantly underestimate the contribution of marine invertebrates to dog diet.

**Table 1:** Estimated Broken Group Island dog diet (n = 14) showing percentage contributions at low (25%), midpoint (50%) and high (75%) ranges for dietary contributions based on mixing model outputs.

Model	Shellfish			Salmon and forage fish			Marine mammal			Nearshore fish			Terrestrial mammal		
	Low	Mid	High	Low	Mid	High	Low	Mid	High	Low	Mid	High	Low	Mid	High
A1	14.8	19.3	24.0	11.2	20.8	28.8	20.8	27.7	34.7	13.2	26.4	38.2	4.2	6.0	7.8
B1	20.7	24.4	27.6	36.3	40.4	45.0	19.7	25.5	30.1	3.9	8.7	15.4	–	–	–
C1	–	–	–	55.2	60.1	65.4	2.2	4.8	8.3	27.8	34.4	40.2	–	–	–

### Spatial and temporal variation among sites within the Broken Group Islands

**Figure 5:**
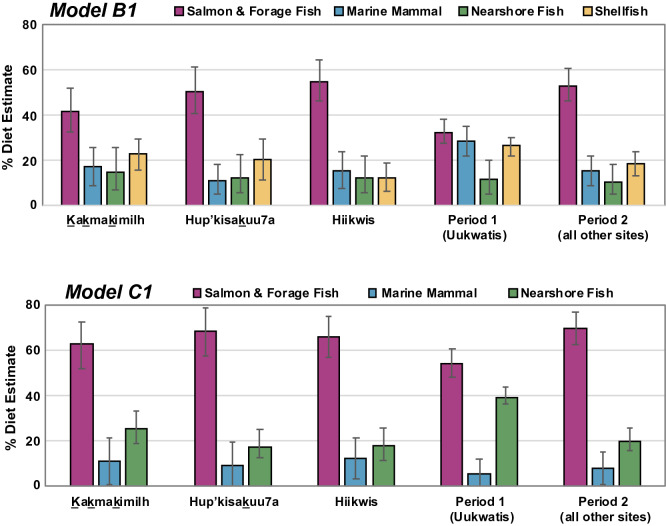
Estimated contributions of food items to Broken Group Island (Tseshaht territory) dog diet (n = 14) by archaeological site and temporal period, as represented by models B1 (top) and C1 (bottom; Supplementary Table S5 for numerical output). Error bars represent 25 and 75% quartile ranges.

A comparison of dietary patterns among the three contemporaneous sites (1,200–300 cal BP) and one older deposit (2,880–1830 cal BP) suggests modest spatial and temporal variation at a local scale (Fig. 5). Site specific results from model B1 indicate that the two dogs from Kakmakimilh were generalist consumers with moderately greater use of salmon/forage fish but with considerable overlap in the interquartile range between shellfish, marine mammals and nearshore fish. Similarly, results from the nearby island site of Hup’kisakuu7a suggests a diet largely composed of salmon/forage fish, followed by shellfish and more distantly by nearshore fish and marine mammals. The two dogs from Hiikwis (on Vancouver Island) had a diet with greater focus on salmon/forage fish with marine mammals a distant second, followed by shellfish and nearshore fish. Older deposits at Uukwatis (2,880–1,830 cal BP), less than 1 km away, showed relatively equal use of food groups.

In contrast, results from model C1 (excluding shellfish) consistently indicated that dog diets at all three late period sites exhibited specialist diets focused on salmon/forage fish with median values between 62–77%, with only modest contributions from nearshore fish and marine mammals across all sites and time periods (Fig. 5). This pattern extends to the oldest sampled dogs from Uukwatis, which had a higher percent of nearshore fish but relatively similar salmon/forage fish values over this broad time range. Notably both models B1 and C1 indicated a higher percent of nearshore fish at the oldest deposit at Uukwatis (see Supplementary Table S5).

### Zooarchaeological assemblage data

**Figure 6:**
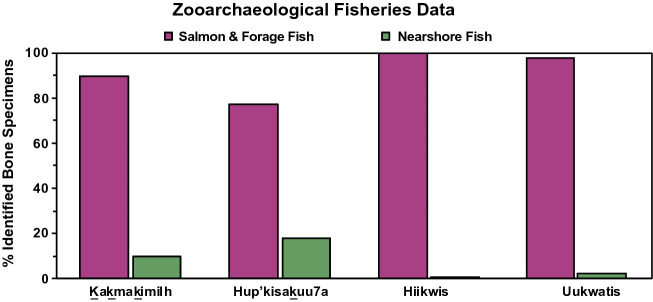
Relative number of identified specimens (NISP) of marine fish recovered from fine screen (2 mm) zooarchaeological deposits for each site (n = 4) grouped according to MixSIAR categories.

A considerable range of marine animals were present in zooarchaeological assemblages from the four sites including birds, mammals, fish, the latter being the most overwhelmingly abundant vertebrate. Column sample analysis of all bones larger than 2 mm showed that between 75–98% of identified vertebrate remains across the four sites were composed of salmon/forage fish followed distantly by nearshore fish (Fig. 6). Together, anchovy and herring account for 99% of the number of identified specimens (NISP) in column samples at Hiikwis, 98% at Uukwatis, 90% at Kakmakimilh, and 77% at Hup’kisakuu7a. This taxonomic composition is consistent with other sites in Barkley Sound^38,45^ and informed the formation of model categories, which were based on the rank order prevalence within site assemblages and where isotope samples were obtained. These zooarchaeological data demonstrate that there was abundant harvested marine biomass which dogs could be provisioned and/or consumed via scavenging on the by-products of processing, storage, and consumption by humans.

## Discussion

We integrated stable isotope analysis, isotopic modelling, and quantitative zooarchaeological data to gain insight into domestic dog diets, and by extension, their human companions in areas of the Pacific Northwest Coast during the late Holocene. Our analysis of isotopic values from 14 domestic dogs and numerous potential foods from Tseshaht territory in Barkley Sound provides new evidence documenting how dog diets varied considerably across the coast with more subtle variation within the Broken Group Islands and over time. Irrespective of which candidate model was examined, variation in Broken Group Islands is broadly consistent over three millennia. Over this period, such patterns indicate an overwhelmingly marine diet with a prominent role of salmon/forage fish with more diverse emphasis on marine mammal and shellfish, and modest use of nearshore fish in model B1. At the coarse scale, the proportions of major food groups estimated by model B1 suggests dogs in Broken Group Islands, Tseshaht territory, had more generalist diets, but at a finer scale the C1 model suggests the likelihood that dog diet was more specialized on salmon and forage fish (representing nearly two thirds of dog diet). Recognizing the limitations of a mixing model approach and our analytical resolution across multiple food sources, we interpret both model B1 and C1 results as alternative hypotheses that complement one another by providing different levels of analytical resolution into a clearly intensive marine diet.

In evaluating the weight of evidence for each model, we draw from zooarchaeological bone count data and contextual ecological and archaeological information to estimate the contributions of various foods. These mixing models are broadly consistent with zooarchaeological abundance data where the majority of faunal assemblages are composed of marine fishes and indicate support for models B1 and C1. Together, these complementary datasets indicate dogs are both directly and indirectly provisioned with abundant marine protein. Notably, there is a very high proportion of salmon and forage fish (i.e., northern anchovy & Pacific herring) in the archaeological record in Broken Group Islands and in the four study sites in particular. Together, salmon and forage fish accounted for over 99% of all identified vertebrate specimens larger than 2 mm at Hiikwis, 98% at Uukwatis, 90% at Kakmakimilh, and 77% at Hup’kisakuu7a (Fig. 6). In contrast, results for model B1 suggest that shellfish comprised up to a quarter of dog diet across sites and time periods. Yet when shellfish was excluded in the C1 model, dogs appeared to exhibit specialization on salmon/forage fish with modest levels of nearshore fish and reduced use of marine mammals. Whereas shellfish comprise a significant proportion of archaeological deposits in the study area^23,46,47^ these small and individually processed food items require substantial human effort to enable dog consumption making it less likely people were provisioning dogs with substantial amounts of shellfish. It is also possible that dogs opportunistically consumed non-burrowing invertebrates in the rocky intertidal, but it is doubtful this would constitute a large proportion of their diet.

The consistency of mixing model results for salmon/forage fish, in addition to multiple lines of evidence, support the interpretation that these taxa comprised common and important foods for dogs in the Broken Group Islands, Tseshaht territory, resources ultimately acquired by humans. Notably, the greatest spawning aggregations for herring on western Vancouver Island observed throughout the twentieth century occur in west Barkley Sound^48^ and directly adjacent to the sites of Hiikwis and Uukwatis. This latter site has a Nuu-chah-nulth place name “herring-guts on-the-rocks”^49^. Further evidence for a past abundance of forage fish is documented in marine sediment cores from nearby Effingham Inlet (a sheltered inlet in Barkley Sound), where both herring and anchovy are the two most abundant fish spanning the last 2,800 years^50^. Clearly, Tseshaht First Nations ancestors were harvesting significant quantities of these fish, a portion of which could then be consumed by their dogs. Whereas the degree to which dogs were selectively provisioned versus consuming secondary waste remains unclear, it seems plausible to purposefully feed dogs a portion of a larger catch, given the substantial effort required to prepare fish for longer term storage^51,52^.

Ethnographic sources also provide value to our interpretation. Records noted that dogs “lived on what scraps they could scavenge, chiefly offal”^21^ indicating an abundance of food processing by-products, most of which are fish (Fig. 6). This account also emphasized that dogs were not permitted to eat salmon during the early part of the salmon runs, which is consistent with similar ‘first salmon’ taboos elsewhere on the coast^14,53^. An additional consideration for uncooked salmon is salmon poisoning disease (SDP), a disease agent prevalent in this region that can be fatal for canids^54^. However, anthropologists and Indigenous knowledge holders have argued that preindustrial dogs likely exhibited immunity to this disease^14,55^. Moreover, the disease has not yet been documented in modern salmon eating coastal wolf populations^56^, which selectively consume salmon heads^57^. Local salmon spawning populations of all five marine salmon species occur in Barkley Sound. Based on studies elsewhere, salmon populations were likely much larger prior to twentieth century industrial fishing^58,59^. Prior to contact, these fish could also be harvested in large quantities using mass capture techniques (e.g., nets, rakes, & fish traps)^21^ and processed for long term storage, so they could have provided a reliable source of food and trade over the year^60^.

Surprisingly, use of terrestrial mammals, such as deer, was nearly absent in our model outputs. These animals are represented, however, in the zooarchaeological record, which informed our initial inclusion of these items in model A1. However, the results of that model suggest only minimal consumption (6%, 4–8%; Table 1) and informed our decision to consider alternative dietary scenarios such as the marine-only B and C dietary models.

Similarly, despite the high-quality habitat and consistent presence of nearshore fish remains in proximity to the study sites and in the archaeological assemblages, MixSIAR results for candidate models (B1 and C1) indicate that Broken Group Island dogs consumed only modest amounts of nearshore fish (up to 9% and 34% under models B1 and C1, respectively). Such a pattern contrasts with documentation of their clear value for human harvesting^61^. It is also possible that these taxa were purposefully discouraged for use in provisioning dogs, as the long-lived genus *Sebastes* sp. have particularly robust bones and dorsal spines, which could pose consumption hazards (e.g., an esophagus or intestinal puncture).

A continued source of uncertainty in our interpretation of MixSIAR outputs, as in all stable isotope mixing model approaches, is the limited number of potential food groups, particularly for generalist consumers such as canids. Recent modelling efforts have noted the challenge of predicting certain prey sources when contrasted with direct feeding observations in aquatic environments^62^. We acknowledge that the configuration of isospace represented in our mixing model scenarios has the potential for excluded dietary items (that were actually used, even if proportionally minor) to change dietary source estimates. Likewise, existing categories could shift with the inclusion of additional specimens and species. We anticipate future modelling approaches will refine food source estimates and apply them to different coastal regions. Earlier studies have relied on direct comparisons of isotopic values and prey sources that have had trophic discrimination factors applied. Considering our data using this more straightforward visual analysis further supports our modelling observations in that dog values most closely overlap with salmon and forage fish when corrected for trophic enrichment (Fig. 2).

On a broader coast-wide scale, stable isotope compositions reflect economic practices of the dogs’ human companions including large-scale ecological and environmental gradients. Whereas we are unable to produce model estimates from all sites across the Northwest Coast due to potential differences in isotopic baselines, visual comparison of dog values reveals that the Broken Group Islands in Tseshaht territory are isotopically unique with both elevated *δ*^13^C and *δ*^15^N (Fig. 3). This distinct isotopic position likely indicates ecological differences in food consumption as well as culturally distinct patterns of resource use and provisioning of dogs. Although it is clear that all dogs extensively used marine derived nutrients, those from the interior regions consumed much more terrestrial foods than all other populations. On the other hand, dogs from outer coast locations, including the Broken Group, Gwaii Hannas, and Port Hardy (Fig. 3), demonstrate overwhelming use of marine resources, likely including species acquired in pelagic and kelp-rich settings. This comparison provides an understanding of how dog diets, and ultimately human resource use, can be illuminated across an isotopic gradient of inland to exposed outer coast locations.

Although specific to an area of the Pacific Northwest Coast, this study has relevance for interpreting variability in dog diets more broadly. On a global scale, direct comparison of isotopic values is problematic because baseline isotopic data are often lacking. However, comparison with prior studies of dog diets in the North Pacific indicate similar focus on marine resources. For instance, dogs in two areas of coastal Alaska appear to be provisioned with Pacific salmon as well as other marine foods including Pacific cod and marine mammals^63,64^. Elsewhere, a diet of nearshore marine fish and marine mammals is documented in Southern California’s Channel Islands^65^ and in northern Hokkaido, Japan^66^. Collectively, such patterns indicate similarly widespread use of marine resources to provision coastal dogs but highlights the elevated role of forage fish in Tseshaht dog diets. This latter protein source appears unique, and associated inference suggests that mass capture technologies supported dog husbandry practices in this region. We expect future work will refine dietary estimates and expand baseline data, and thus, provide greater insight into specific food provisioning practices for dogs on the Northwest Coast and elsewhere in the world.

## Conclusions

Combining stable isotope analysis of dog bone collagen with zooarchaeological evidence provides detailed insight into historic food web activities and past ecological conditions. By applying mixing model methods to established comparisons of stable isotope plot associations, along with site-specific zooarchaeological data, we were able to provide direct and detailed evidence for the consumption of marine resources by dogs and humans of Tseshaht territory. This study contributes to research in other regions where preindustrial canid diets have been isotopically examined using mixing models^64,67,68^. On a regional scale, these findings provide insight into dog provisioning practices across the Northwest Coast, as well as how past people interacted with the environment around them. Such perspective allows us to better understand human–dog relationships, animal husbandry practices and the economic importance these dogs had in Tseshaht Territory and beyond.

## Supplementary information

Supplementary Information 1.

## Data Availability

Annotated R code and isotopic data are provided in the supporting information.
