# Supplementary material for: Ancient dog diets on the Pacific Northwest Coast: zooarchaeological and stable isotope modelling evidence from Tseshaht territory and beyond
Source: Sci Rep. 2020 Oct 1;10:15630. doi: 10.1038/s41598-020-71574-x (PMC7530995; doi:10.1038/s41598-020-71574-x)
Supplement: Supplementary file 1 — Supplementary Information 1. [file 41598_2020_71574_MOESM1_ESM.pdf]

Supplementary Information for:

Ancient dog diets on the Pacific Northwest Coast: zooarchaeological and stable isotope modelling evidence from Tseshaht territory and beyond

Dylan Hillis, Iain McKechnie, Eric Guiry, Denis E. St. Claire, & Chris T. Darimont

Scientific Reports DOI: 10.1038/s41598-020-71574-x

Supplementary Information Document S1

Stable Isotope Measurement and Calibration

Stable carbon and nitrogen isotope compositions were calibrated relative to Vienna Pee Dee Belemnite (VPDB) and atmospheric nitrogen (AIR), respectively. The integrity of stable isotope measurements was assessed using the following criteria: 1) bone collagen yield above 2%; 2) atomic carbon/nitrogen ($C{:}N_{Atomic}$) ratio between 2.9 and 3.6, and; 3) elemental concentrations above 13% and 4.8% for carbon and nitrogen, respectively [1-3].

For collagen samples analyzed in the Archaeology Chemistry Laboratory at the University of British Columbia (UBC), samples were calibrated using a two-point calibration curve anchored to glutamic acid standards, USGS40 ($\delta^{13}C$ = – 26.39 ‰; $\delta^{15}N$ = – 4.52 ‰) and USGS41 ($\delta^{13}C$ = + 36.55 ‰; $\delta^{15}N$ = + 47.57 ‰) or USGS41a ($\delta^{13}C$ = – 36.55 ‰; $\delta^{15}N$ = + 47.55 ‰). Uncertainty was monitored using several in-house standards with well-characterized isotopic compositions: methionine (MET, $\delta^{13}C$ – 28.62 ± 0.11 ‰ and $\delta^{15}N$ – 5.03 ± 0.15 ‰), SRM-1 (caribou bone collagen, $\delta^{13}C$ – 19.36 ‰, $\delta^{15}N$ + 1.81 ‰), SRM-2 (walrus bone collagen, $\delta^{13}C$ – 14.76 ‰, $\delta^{15}N$ + 15.59 ‰), SRM-14 (polar bear bone collagen, $\delta^{13}C$ – 13.66 ‰, $\delta^{15}N$ + 21.63 ‰), SRM-15 (deer bone collagen, $\delta^{13}C$ – 26.80 ± 0.01 ‰, $\delta^{15}N$ + 6.90 ± 0.04 ‰), SRM-16 (seal bone collagen, $\delta^{13}C$ – 14.80 ± 0.03 ‰, $\delta^{15}N$ + 16.91 ± 0.05 ‰). Sample precision was determined to be ± 0.05 ‰ for $\delta^{13}C$ and ± 0.07 ‰ $\delta^{15}N$ on the basis of repeated measurements of calibration and check standards. Analytical accuracy was determined to be ± 0.05 ‰ for $\delta^{13}C$ and ± 0.08 ‰ for $\delta^{15}N$ following Szpak et al. [4].

At the Department of Soil Science Isotope Lab at the University of Saskatchewan (USask), uncertainty was determined with one in-house collagen standard: Ref/EM Protein 1 (Elemental Microanalysis (cat. B2155, $\delta^{13}C$ – 27.15 ± 0.08 ‰, $\delta^{15}N$ + 5.97 ± 0.04 ‰). Uncertainty between stable isotope facilities was monitored using two UBC in-house collagen standards: SRM-15 (deer bone collagen, $\delta^{13}C$ – 26.80 ‰, $\delta^{15}N$ + 6.90 ‰), SRM-16 (seal bone collagen, $\delta^{13}C$ – 14.80 ‰, $\delta^{15}N$ + 16.91 ‰). Based on repeated measurements of SRM-15 and SRM-16 at USask, the isotopic composition was determined to be: SRM-15 ($\delta^{13}C$ – 26.73 ± 0.08 ‰, $\delta^{15}N$ + 6.72 ± 0.03 ‰) and SRM-16 ($\delta^{13}C$ – 14.91 ± 0.17 ‰, $\delta^{15}N$ + 16.66 ± 0.03 ‰). For collagen samples analyzed at USask, sample precision was determined to be ± 0.09 ‰ for $\delta^{13}C$ and ± 0.22 ‰ $\delta^{15}N$ on the basis of repeated measurements of UBC in-house standards. Analytical accuracy was determined to be ± 0.03 ‰ for $\delta^{13}C$ and ± 0.05 ‰ for $\delta^{15}N$.

At the Department of Plant Sciences Stable Isotope Facility at the University of California Davis (UC Davis), collagen samples were calibrated using a two-point calibration curve anchored to glutamic acid standards, USGS40 ($\delta^{13}C$ = – 26.39 ‰; $\delta^{15}N$ = – 4.52 ‰) and USGS41a ($\delta^{13}C$ = – 36.55 ‰; $\delta^{15}N$ = + 47.55 ‰). Uncertainty was monitored using four UBC in-house standards with well-characterized isotopic compositions. Sample precision was determined to be ± 0.20 ‰ for $\delta^{13}C$ and ± 0.12 ‰ $\delta^{15}N$ on the basis of repeated measurements of UBC in-house standards (USGS40, USGS41a, MET and SRM-15). Analytical accuracy was determined to be ± 0.34 ‰ for $\delta^{13}C$ and ± 0.08 ‰ for $\delta^{15}N$. Uncertainty between stable isotope facilities was monitored using four UBC in-house collagen standards: USGS40 ($\delta^{13}C$ = – 26.39 ‰; $\delta^{15}N$ = – 4.52 ‰), USGS41a ($\delta^{13}C$ = – 36.55 ‰; $\delta^{15}N$ = + 47.55 ‰), methionine (MET, $\delta^{13}C$ – 28.62 ‰ and $\delta^{15}N$ – 5.03 ‰) and SRM-15 (deer bone collagen, $\delta^{13}C$ – 26.80 ‰, $\delta^{15}N$ + 6.90 ‰). Based on repeated measurements of USGS40, USGS41a, MET and SRM-15 at UC Davis, the isotopic composition was determined to be: USGS40 ($\delta^{13}C$ = – 26.36 ± 0.04 ‰; $\delta^{15}N$ = – 4.43 ± 0.07 ‰), USGS41a ($\delta^{13}C$ = – 37.26 ± 0.13 ‰; $\delta^{15}N$ = + 47.3 ± 0.38 ‰), MET ($\delta^{13}C$ – 28.60 ± 0.05 ‰, $\delta^{15}N$ – 4.95 ± 0.09 ‰) and SRM-15 ($\delta^{13}C$ – 26.86 ± 0.05 ‰, $\delta^{15}N$ + 6.84 ± 0.08 ‰).

Supplementary Information: Tables

Table S1. Radiocarbon results for archaeological deposits associated with domestic dogs used in this study.*

Site	Unit	Level	Dog Sample Number **	^{14}C Lab no.	Material	^{14}C age (BP)	Calibrated age-range***	Source
93T (DfSh-43) (Hup'kisakuu7a)	2	D3	6540	D-AMS 013550	Charcoal	720±20	685–650	Smith et al. 2015 [5]
306T (DfSh-17) (Kakmakimilh)	8	D3	4149 & 6538	UOC-5838	Deer	950±40	930–745	Kieser 2018 [6]
306T (DfSh-17) (Kakmakimilh)	8	D3	4149 & 6538	UOC-11932	Charcoal	650±26	670-555	Kieser 2020 [7]
306T (DfSh-17) (Kakmakimilh)	8	D3	4149 & 6538	UOC-11925	Charcoal	682±26	675-560	Kieser 2020 [7]
DfSh-16 (Hiikwis)	N4-6/ E0-2	A7	6977	Beta-265975	Charcoal	380±50	510–315	MacLean 2012:61 [8]
DfSh-16 (Hiikwis)	N4-6/ E0-2	B13	6974	Beta-250331	Charcoal	880±40	910–690	MacLean 2012:61 [8]
DfSh-15 (Uukwatis)	4A	A4	6972	Beta-250324	Charcoal	2,020±40	2,100–1,834	MacLean 2012:55 [8]
DfSh-15 (Uukwatis)	4A	B10	6973	Beta-250326	Charcoal	2,210±40	2,335–2,120	MacLean 2012:55 [8]
DfSh-15 (Uukwatis)	4	6	6978	Beta-250324	Charcoal	2,020±40	2,100–1,834	MacLean 2012:55 [8]
DfSh-15 (Uukwatis)	4	7	6980	Beta-250324	Charcoal	2,020±40	2,100–1,834	MacLean 2012:55 [8]
DfSh-15 (Uukwatis)	4	10	6976	Beta-250326	Charcoal	2,210±40	2,335–2,120	MacLean 2012:55 [8]
DfSh-15 (Uukwatis)	4	22	6983	Beta-250330	Charcoal	2,710±40	2,880–2,750	MacLean 2012:55 [8]

* Note: some levels from which dogs were recovered lack level-specific radiocarbon dates.
** See Table S6.
*** Calibrated age-ranges are in calendar years before present at 2-sigma probability (95.4%) and were calibrated with INTCAL20[9] using Oxcal[10] and rounded to the nearest 5 year interval.

Table S2. Source samples were supplemented with 166 previously published isotopic values of potential prey specific to the west coast of Vancouver Island and the Olympic Peninsula.

Food Group	Taxa	*n*	Reference
Marine Mammal	*Eschrichtius robustus* (Gray Whale)	30	Alter et al. 2012 [11]
	Callorhinus ursinus (Northern fur seal)	62	Newsome et al. 2007 [12]
	Phoca vitulina (Harbour seal)	55	Newsome et al. 2007 [12]
Shellfish*	*Mytilus californianus* (California mussel) *	19	Markel 2011[13]; Markel & Shurin 2015 [14]

* Shellfish values are from modern tissue (see Table S4).

Table S3. Taxonomic composition of the 5 evaluated food groups for the *MixSIAR* model scenarios based on isotope values for western Vancouver Island and the Olympic Peninsula.

Food Group	Taxa	Isotope Samples
**Salmon & Forage Fish**	*Oncorhynchus sp.* (Salmon sp.), *Clupea pallasii* (Pacific herring), and *Engraulis mordax* (Northern anchovy)	56
**Marine Mammal**	*Callorhinus ursinus* (Northern fur seal), *Eschrichtius robustus* (Gray whale) and *Phoca vitulina* (Harbour seal)	167
**Shellfish**	*Mytilus californianus* (California mussel)	19
**Nearshore Fish**	*Embiotocidae sp.* (Perch), *Hexagrammos sp.* (Greenling), *Ophiodon elongatus* (Lingcod), *Sebastes sp.* (Rockfish) and *Squalus suckleyi* (Dogfish)	59
**Terrestrial Mammal**	*Odocoileus hemionus* (Black-tailed deer)	6

Table S4. Food source categories used in *MixSIAR* modelling. Categories represent isotopically distinct ecological-based categories, which are specific to western Vancouver Island and the Olympic Peninsula.

Diet	$\delta^{13}C$	SD	$\delta^{15}N$	SD	*n*	Sample Type
Terrestrial Mammal	-22.7	1.4	3.3	0.9	6	Late-Holocene deer bone collagen
Shellfish*	-11.7	0.4	9.0	0.4	19	Modern tissue [13,14]
Salmon & Forage Fish	-13.9	1.4	13.1	1.5	56	Late-Holocene fish bone collagen
Marine Mammal	-12.9	0.7	17.1	0.8	167	Late-Holocene marine mammal bone collagen
Nearshore Fish	-11.7	1.0	14.6	1.2	59	Late-Holocene fish bone collagen

**Shellfish Assumptions: (1) $\delta^{13}C$ collagen = $\delta^{13}C$ tissue + 3.7 ‰, (2) $\delta^{15}N$ collagen = $\delta^{15}N$ tissue, (3) $\delta^{13}C$ late-Holocene tissue = $\delta^{13}C$ modern tissue + 1 ‰.*

Table S5. Estimated contribution of food items to dog diets in the Broken Group Islands, Tseshaht territory, village sites (local scale) and change over time (temporal scale).

Population		Shellfish			Salmon & Forage Fish			Marine Mammal			Nearshore Fish		
**K̲ak̲mak̲imilh** (*n* = 2)	**B1**	15.4	**23.0**	29.4	32.2	**41.3**	51.7	8.8	**16.9**	25.4	6.7	**14.9**	25.7
	**C1**	-	-	-	52.0	**62.6**	72.8	5.3	**11.4**	19.3	13.4	**25.2**	38.4
**Hup'kisak̲uu7a** (*n* = 1)	**B1**	11.2	**20.6**	29.4	40.4	**50.5**	61.3	5.0	**10.7**	18.3	5.5	**12.3**	22.6
	**C1**	-	-	-	57.3	**68.3**	78.5	4.3	**9.5**	17.1	8.4	**17.7**	30.4
**Hiikwis** (*n* = 2)	**B1**	6.4	**12.2**	18.5	46.2	**54.9**	64.1	7.8	**15.4**	23.9	5.4	**12.4**	22.0
	**C1**	-	-	-	57.0	**65.8**	75.1	5.9	**12.3**	20.3	8.9	**18.0**	29.0
**Period 1** (Uukwatis) (*n* = 9)	**B1**	21.9	**26.3**	30.2	27.4	**32.4**	37.8	21.6	**28.6**	34.7	5.1	**11.4**	19.9
	**C1**	-	-	-	48.1	**54.3**	60.7	2.5	**5.4**	9.6	31.6	**39.4**	46.6
**Period 2** (Hiikwis, Hup'kisak̲uu7a and K̲ak̲mak̲imilh) (*n* = 5)	**B1**	12.9	**18.6**	23.7	46.2	**52.9**	60.4	8.8	**15.5**	22.0	4.8	**10.5**	18.3
	**C1**	-	-	-	62.6	**69.6**	76.9	3.6	**7.9**	13.7	11.7	**20.2**	29.2

Note: all results are presented as the 25%, 50% and 75% percentile estimate.

Table S6. Stable isotope values for samples used in *MixSIAR* modelling. Included are UBC sample number, the site location, the site number, the taxonomic classification of the sampled organism, the element analyzed, the δ^{13}C and δ^{15}N isotope values, the percent carbon and percent nitrogen values, and the carbon/nitrogen ratio.

Sample Num.	Site Name	Site Number	Taxon	Element	δ^{13}C	δ^{15}N	%C	%N	C:N
4459	Huts'atswilh	83T	*Callorhinus ursinus*	Not recorded	-14.9	18.3	42.6	14.8	3.4
4460	Huts'atswilh	129T	*Callorhinus ursinus*	Not recorded	-15.0	15.0	37.4	13.4	3.3
4527	Mak̲tl7̲ii	206T	*Callorhinus ursinus*	Not recorded	-15.0	17.8	39.0	13.0	3.5
4531	Shiwitis	82T	*Callorhinus ursinus*	Not recorded	-14.8	17.8	42.6	14.4	3.5
4546	K̲ak̲mak̲imilh	306T	*Callorhinus ursinus*	Not recorded	-15.2	17.3	25.0	8.6	3.4
6525	K̲ak̲mak̲imilh	306T	*Callorhinus ursinus*	Not recorded	-14.5	16.8	38.0	13.0	2.9
6782	Ts'ishaa	204T	*Callorhinus ursinus*	Rib	-12.3	18.6	41.6	14.5	2.9
5 UC	K̲ak̲mak̲imilh	306T	*Callorhinus ursinus*	Not recorded	-14.2	18.0	20.6	6.7	3.1
4149 D	K̲ak̲mak̲imilh	306T	*Canis familiaris*	Ulna	-11.8	16.3	41.7	15.0	3.2
4150 D	K̲ak̲mak̲imilh	306T	*Canis familiaris*	R. 2nd Rib	-11.9	16.9	41.1	14.6	3.3
6540*	Hup'kisak̲uu7̲a	93T	*Canis familiaris*	Metacarpal	-12.7	16.1	41.5	14.6	3.3
6969*	Uukwatis	DfSh-15	*Canis familiaris*	L. Mandible	-11.6	17.2	40.4	14.5	3.3
6970*	Uukwatis	DfSh-15	*Canis familiaris*	Proximal Rib	-11.7	17.4	40.7	14.8	3.2
6972*	Uukwatis	DfSh-15	*Canis familiaris*	Cervical Vertebrae	-12.9	15.9	40.4	14.5	3.2
6975*	Uukwatis	DfSh-15	*Canis familiaris*	L. Rib	-11.4	16.8	40.7	14.8	3.2
6976*	Uukwatis	DfSh-15	*Canis familiaris*	Baculum	-11.8	16.9	41	15	3.2
6977*	Hiikwis	DfSh-16	*Canis familiaris*	R. Uniciform (Hamate)	-12.6	16.5	40.9	14.9	3.2
6979*	Uukwatis	DfSh-15	*Canis familiaris*	L. Proximal Tibia	-11.8	17.2	40.4	14.7	3.2
6980*	Uukwatis	DfSh-15	*Canis familiaris*	Auditory bulla/zygomatic process of squamosal	-11.6	16.7	40.8	14.8	3.2
6983*	Uukwatis	DfSh-15	*Canis familiaris*	R. Proximal Radius	-12.4	17.4	40.2	14.6	3.2
6974*	Hiikwis	DfSh-16	*Canis familiaris*	Thoracic Vertebrae	-13.0	17.5	40.7	14.4	3.3
6973*	Uukwatis	DfSh-15	*Canis familiaris*	Proximal Rib	-12.1	16.9	41	14.9	3.2
6538* D	K̲ak̲mak̲imilh	306T	*Canis familiaris*	R. 4th Rib	-12.5	16.5	41.6	15.0	3.2
6539* D	K̲ak̲mak̲imilh	306T	*Canis familiaris*	Carpal (scapholunar)	-12.6	16.7	41.1	14.7	3.3
4155	K̲ak̲mak̲imilh	306T	*Clupea pallasii*	Vertebrae	-12.6	13.2	28.8	10.2	3.3
4160	K̲ak̲mak̲imilh	306T	*Clupea pallasii*	Vertebrae	-13.1	13.3	33.9	12.0	3.3
4170	K̲ak̲mak̲imilh	306T	*Clupea pallasii*	Not recorded	-13.2	14.0	34.5	12.0	3.3
4172	K̲ak̲mak̲imilh	306T	*Clupea pallasii*	Vertebrae	-13.5	13.2	34.9	12.2	3.3
4177	K̲ak̲mak̲imilh	306T	*Clupea pallasii*	Vertebrae	-13.5	13.3	33.4	11.6	3.4
4189	K̲ak̲mak̲imilh	306T	*Clupea pallasii*	Vertebrae	-12.7	13.1	34.3	12.1	3.3
4192	K̲ak̲mak̲imilh	306T	*Clupea pallasii*	Vertebrae	-13.5	14.1	38.4	13.5	3.3

Sample Number	Site Name	Site Number	Taxon	Element	$\delta^{13}C$	$\delta^{15}N$	%C	%N	C:N
4197	Kakmakimilh	306T	*Clupea pallasii*	Vertebrae	-13.3	13.1	33.6	12.0	3.3
4210	Kakmakimilh	306T	*Clupea pallasii*	Vertebrae	-13.6	14.5	34.3	11.9	3.4
4440	Huts'atswilh	83T	*Clupea pallasii*	Unknown	-13.1	12.8	42.2	14.8	3.3
4441	Huts'atswilh	83T	*Clupea pallasii*	Unknown	-13.0	12.9	43.4	15.1	3.4
4442	Huts'atswilh	129T	*Clupea pallasii*	Not recorded	-12.6	13.1	42.8	15.8	3.2
6379	Hup'kisakuu7a	93T	*Clupea pallasii*	Not recorded	-13.3	14.0	40.6	14.5	3.3
6381	Kakmakimilh	306T	*Clupea pallasii*	Cranial	-13.7	14.5	32.9	11.2	2.9
6383	Hup'kisakuu7a	93T	*Clupea pallasii*	Vertebrae	-14.1	11.9	26.3	8.9	2.9
4451	Huts'atswilh	129T	*Embiotocidae*	Not recorded	-12.1	12.8	42.0	15.1	3.2
4205	Kakmakimilh	306T	*Embiotocidae*	Vertebrae	-11.8	13.6	41.6	14.5	3.3
4187	Kakmakimilh	306T	*Embiotoca lateralis*	Vertebrae	-11.3	12.8	36.3	13.2	3.2
6449	Kakmakimilh	306T	*Embiotocidae*	Unknown	-8.8	14.6	37.9	12.9	3.4
6450	Kakmakimilh	306T	*Embiotocidae*	Unknown	-11.8	12.8	38.8	13.4	3.4
6451	Kakmakimilh	306T	*Embiotocidae*	Vertebrae	-10.1	14.4	34.9	11.7	3.5
6452	Kakmakimilh	306T	*Embiotocidae*	Hyomandibular	-11.0	13.7	39.0	13.6	3.3
6453	Kakmakimilh	306T	*Embiotocidae*	Not recorded	-11.4	13.5	38.8	13.8	3.3
4154	Kakmakimilh	306T	*Engraulis mordax*	Vertebrae	-12.5	12.0	25.2	8.6	3.4
4161	Kakmakimilh	306T	*Engraulis mordax*	Vertebrae	-12.4	11.4	39.0	14.2	3.2
4173	Kakmakimilh	306T	*Engraulis mordax*	Vertebrae	-12.8	11.3	31.5	11.3	3.2
4183	Kakmakimilh	306T	*Engraulis mordax*	Vertebrae	-12.7	12.0	37.0	13.5	3.2
4193	Kakmakimilh	306T	*Engraulis mordax*	Vertebrae	-13.8	11.9	31.0	11.1	3.3
4206	Kakmakimilh	306T	*Engraulis mordax*	Vertebrae	-13.0	12.4	35.9	12.9	3.3
6386	Hup'kisakuu7a	93T	*Engraulis mordax*	Vertebrae	-13.6	11.8	30.8	10.7	2.9
6387	Huts'atswilh	83T	*Engraulis mordax*	Vertebrae	-13.4	12.4	28.8	9.7	3.0
4153	Kakmakimilh	306T	*Hexagrammos sp.*	Vertebrae	-11.3	13.3	39.2	13.9	3.3
4159	Kakmakimilh	306T	*Hexagrammos sp.*	Vertebrae	-11.1	13.2	30.3	10.6	3.3
4166	Kakmakimilh	306T	*Hexagrammos sp.*	Vertebrae	-11.4	13.6	42.9	15.9	3.1
4171	Kakmakimilh	306T	*Hexagrammos sp.*	Angular	-11.3	13.7	41.1	15.2	3.2
4178	Kakmakimilh	306T	*Hexagrammos sp.*	Not recorded	-11.8	13.9	39.5	14.3	3.2
4185	Kakmakimilh	306T	*Hexagrammos sp.*	Not recorded	-12.6	13.1	42.1	15.5	3.2
4201	Kakmakimilh	306T	*Hexagrammos sp.*	Vertebrae	-11.9	13.7	32.2	11.3	3.3
4521	Huts'atswilh	129T	*Hexagrammos sp.*	Not recorded	-10.4	14.2	42.3	15.5	3.2
6399	Hup'kisakuu7a	93T	*Hexagrammos sp.*	Vertebrae	-11.7	13.2	40.6	14.1	3.3
6400	Kakmakimilh	306T	*Hexagrammos sp.*	Ceratohyal	-12.6	14.4	33.7	11.0	3.1
6401	Kakmakimilh	306T	*Hexagrammos sp.*	Not recorded	-10.3	14.4	39.7	14.3	3.2
6403	Kakmakimilh	306T	*Hexagrammos sp.*	Hyomandibular	-12.9	13.6	39.3	13.2	3.5
6404	Kakmakimilh	306T	*Hexagrammos sp.*	Not recorded	-10.6	14.4	39.3	13.8	3.3

Sample Number	Site Name	Site Number	Taxon	Element	δ ^{13}C	δ ^{15}N	%C	%N	C:N
6405	Kakmakimilh	306T	*Hexagrammos sp.*	Not recorded	-11.9	13.8	40.3	14.0	3.4
4461	Huts'atswilh	83T	*Mysticeti sp.*	Unknown	-14.5	13.7	42.5	15.1	3.3
4520	Huts'atswilh	129T	*Mysticeti sp.*	Unknown	-13.0	14.2	42.7	14.6	3.4
4530	Shiwitis	82T	*Mysticeti sp.*	Unknown	-13.7	15.9	37.8	13.6	3.3
4533	Huts'atswilh	83T	*Mysticeti sp.*	Unknown	-15.2	12.0	42.5	14.3	3.5
6514	Kakmakimilh	306T	*Mysticeti sp.*	Unknown	-13.3	16.3	38.7	13.6	3.3
6515	Huts'atswilh	83T	*Mysticeti sp.*	Unknown	-12.1	15.9	39.8	13.9	3.3
6516	Huts'atswilh	83T	*Mysticeti sp.*	Unknown	-13.7	15.6	40.8	14.4	3.3
6517	Huts'atswilh	83T	*Mysticeti sp.*	Unknown	-16.5	15.3	39.5	13.7	3.4
6518	Maktl7ii	206T	*Mysticeti sp.*	Unknown	-13.3	14.4	32.4	11.3	3.3
4528	Shiwitis	82T	*Odocoileus hemionus*	Not recorded	-22.0	2.3	42.9	15.3	3.3
4529	Shiwitis	82T	*Odocoileus hemionus*	Not recorded	-21.8	2.1	43.4	15.8	3.2
4525	Huts'atswilh	129T	*Odocoileus hemionus*	Not recorded	-23.2	4.5	43.6	15.7	3.2
8 UC	Kakmakimilh	306T	*Odocoileus hemionus*	Not recorded	-23.7	3.6	31.6	10.8	2.9
7 UC	Kakmakimilh	306T	*Odocoileus hemionus*	Not recorded	-20.8	3.8	14.8	5.0	2.9
4151	Kakmakimilh	306T	*Onchoryhchus sp.*	Vertebrae	-13.7	16.0	40.6	13.8	3.4
4156	Kakmakimilh	306T	*Onchoryhchus sp.*	Vertebrae	-15.8	11.0	39.2	14.1	3.2
4182	Kakmakimilh	306T	*Onchoryhchus sp.*	Vertebrae	-12.3	15.4	39.7	14.7	3.2
4452	Huts'atswilh	129T	*Onchoryhchus sp.*	Not recorded	-16.4	11.6	45.2	15.1	3.5
4544	Kakmakimilh	306T	*Onchoryhchus sp.*	Not recorded	-18.0	10.7	42.1	13.6	3.6
6406	Kakmakimilh	306T	*Onchoryhchus sp.*	Vertebrae	-14.6	12.8	39.4	13.8	3.3
6407	Kakmakimilh	306T	*Onchoryhchus sp.*	Vertebrae	-15.3	11.9	37.9	13.2	3.3
6408	Kakmakimilh	306T	*Onchoryhchus sp.*	Vertebrae	-11.4	15.8	38.7	13.8	3.3
6409	Kakmakimilh	306T	*Onchoryhchus sp.*	Vertebrae	-11.4	15.1	41.1	15.0	3.2
6410	Kakmakimilh	306T	*Onchoryhchus sp.*	Vertebrae	-13.0	15.8	39.4	13.9	3.3
6411	Kakmakimilh	306T	*Onchoryhchus sp.*	Vertebrae	-15.1	12.2	37.4	13.2	3.3
6412	Kakmakimilh	306T	*Onchoryhchus sp.*	Vertebrae	-14.5	12.5	41.7	14.6	3.3
6413	Kakmakimilh	306T	*Onchoryhchus sp.*	Vertebrae	-15.5	12.4	39.8	13.9	3.4
6414*	Kakmakimilh	306T	*Onchoryhchus sp.*	Vertebrae	-12.0	15.0	38.5	13.7	3.3
6416	Kakmakimilh	306T	*Onchoryhchus sp.*	Vertebrae	-14.3	13.9	38.2	13.6	3.3
6417	Kakmakimilh	306T	*Onchoryhchus sp.*	Vertebrae	-12.9	15.1	37.5	13.4	3.3
6419	Kakmakimilh	306T	*Onchoryhchus sp.*	Vertebrae	-13.0	15.4	39.3	13.9	3.3
6420	Kakmakimilh	306T	*Onchoryhchus sp.*	Vertebrae	-14.4	12.5	37.3	13.1	3.3
6421	Kakmakimilh	306T	*Onchoryhchus sp.*	Vertebrae	-14.1	14.0	36.5	12.9	3.3
6422	Kakmakimilh	306T	*Onchoryhchus sp.*	Vertebrae	-15.8	11.8	38.6	13.7	3.3
6423	Kakmakimilh	306T	*Onchoryhchus sp.*	Vertebrae	-15.2	11.7	39.8	13.9	2.9
6424	Kakmakimilh	306T	*Onchoryhchus sp.*	Vertebrae	-16.3	10.7	40.5	14.2	3.3

Sample Number	Site Name	Site Number	Taxon	Element	δ ^{13}C	δ ^{15}N	%C	%N	C:N
6425	Kakmakimilh	306T	*Onchoryhchus sp.*	Vertebrae	-15.8	12.1	39.1	13.4	3.4
6426	Kakmakimilh	306T	*Onchoryhchus sp.*	Vertebrae	-13.3	15.0	37.6	13.0	3.4
6427	Kakmakimilh	306T	*Onchoryhchus sp.*	Vertebrae	-13.0	15.1	39.0	13.8	3.3
6428	Huu7ii	DfSh-7	*Onchoryhchus sp.*	Vertebrae	-15.0	12.3	40.0	14.2	3.3
6430	Huu7ii	DfSh-7	*Onchoryhchus sp.*	Vertebrae	-15.8	10.8	40.3	14.4	3.3
6431	Huu7ii	DfSh-7	*Onchoryhchus sp.*	Vertebrae	-14.8	11.6	39.2	14.0	3.3
6432	Huu7ii	DfSh-7	*Onchoryhchus sp.*	Vertebrae	-15.1	12.5	40.2	14.1	3.3
6434	Huu7ii	DfSh-7	*Onchoryhchus sp.*	Vertebrae	-15.5	11.8	40.1	14.1	3.3
6435	Tl'ihuuw'a	CORE 1	*Onchoryhchus sp.*	Vertebrae	-14.9	13.7	37.5	13.3	3.3
6436	Tl'ihuuw'a	CORE 1	*Onchoryhchus sp.*	Vertebrae	-14.5	13.2	37.4	13.4	3.3
6437	Tl'ihuuw'a	CORE 2	*Onchoryhchus sp.*	Vertebrae	-11.8	15.3	37.4	13.1	3.3
4158	Kakmakimilh	306T	*Ophiodon elongatus*	Vertebrae	-10.8	15.2	34.7	12.7	3.2
4165	Kakmakimilh	306T	*Ophiodon elongatus*	Vertebrae	-11.8	16.3	39.9	14.6	3.2
4202	Kakmakimilh	306T	*Ophiodon elongatus*	Vertebrae	-11.8	16.4	38.2	13.8	3.2
4203	Kakmakimilh	306T	*Ophiodon elongatus*	Not recorded	-11.0	16.9	41.1	15.0	3.2
4444	Huts'atswilh	83T	*Ophiodon elongatus*	Not recorded	-11.0	16.1	42.0	15.1	3.2
4453	Huts'atswilh	129T	*Ophiodon elongatus*	Not recorded	-11.9	16.0	41.9	15.4	3.2
4454	Huts'atswilh	129T	*Ophiodon elongatus*	Not recorded	-13.3	16.8	40.4	13.2	3.6
4455	Huts'atswilh	129T	*Ophiodon elongatus*	Not recorded	-13.1	16.0	41.4	13.9	3.5
4457	Maktl7ii	206T	*Ophiodon elongatus*	Not recorded	-11.0	17.3	42.1	16.2	3.0
6396	Kakmakimilh	306T	*Ophiodon elongatus*	Not recorded	-11.9	15.7	37.7	13.2	2.9
6397	Kakmakimilh	306T	*Ophiodon elongatus*	Vertebrae	-12.1	16.6	41.8	15.2	3.2
6398	Kakmakimilh	306T	*Ophiodon elongatus*	Vertebrae	-10.9	17.2	39.9	14.0	3.3
6532	Hup'kisakuu7a	93T	*Phoca vitulina*	Not recorded	-10.8	18.4	40.9	14.8	2.9
3 UC	Kakmakimilh	306T	*Phoca vitulina*	Not recorded	-10.8	17.4	26.1	9.0	2.9
2 UC	Kakmakimilh	306T	*Phoca vitulina*	Not recorded	-14.1	16.2	28.2	9.9	2.9
4152	Kakmakimilh	306T	*Sebastes sp.*	Vertebrae	-11.0	15.1	30.7	11.0	3.3
4157	Kakmakimilh	306T	*Sebastes sp.*	Vertebrae	-13.0	13.7	33.5	12.0	3.3
4167	Kakmakimilh	306T	*Sebastes sp.*	Premaxilla	-13.3	14.1	44.0	15.7	3.3
4174	Kakmakimilh	306T	*Sebastes sp.*	Not recorded	-12.3	14.6	38.6	13.9	3.2
4176	Kakmakimilh	306T	*Sebastes sp.*	Not recorded	-10.6	14.5	41.9	15.8	3.1
4179	Kakmakimilh	306T	*Sebastes sp.*	Vertebrae	-13.2	14.2	34.2	12.5	3.2
4181	Kakmakimilh	306T	*Sebastes sp.*	Vertebrae	-11.4	15.1	44.6	16.6	3.1
4188	Kakmakimilh	306T	*Sebastes sp.*	Vertebrae	-12.7	13.3	36.3	13.2	3.2
4194	Kakmakimilh	306T	*Sebastes sp.*	Vertebrae	-11.0	14.3	34.8	12.5	3.3
4199	Kakmakimilh	306T	*Sebastes sp.*	Not recorded	-10.8	14.1	37.9	13.6	3.2
4204	Kakmakimilh	306T	*Sebastes sp.*	Not recorded	-11.3	14.9	41.6	15.3	3.2
4207	Kakmakimilh	306T	*Sebastes sp.*	Cleithrum	-10.5	15.5	41.1	14.9	3.2

Sample Number	Site Name	Site Number	Taxon	Element	δ^{13}C	δ^{15}N	%C	%N	C:N
4208	Kakmakimilh	306T	*Sebastes sp.*	Vertebrae	-11.9	14.5	37.7	13.7	3.2
4209	Kakmakimilh	306T	*Sebastes sp.*	Vertebrae	-11.6	16.7	37.8	13.6	3.2
4443	Huts'atswilh	83T	*Sebastes sp.*	Not recorded	-10.8	15.8	42.7	15.1	3.3
4536	Kakmakimilh	306T	*Sebastes sp.*	Not recorded	-13.2	13.4	42.9	14.7	3.4
6392	Kakmakimilh	306T	*Sebastes sp.*	Vertebrae	-13.5	13.6	42.2	14.7	3.3
6394	Kakmakimilh	306T	*Sebastes sp.*	R. Dentary	-13.2	15.0	41.5	14.7	3.3
6395*	Kakmakimilh	306T	*Sebastes sp.*	Not recorded	-11.4	16.1	43.2	15.5	3.2
4168	Kakmakimilh	306T	*Squalus suckleyi*	Vertebrae	-12.8	14.0	41.3	14.1	3.4
4537	Kakmakimilh	306T	*Squalus suckleyi*	Not recorded	-12.3	14.8	42.1	13.8	3.6
6443	Kakmakimilh	306T	*Squalus suckleyi*	Vertebrae	-12.3	14.8	41.8	13.8	3.5
6444	Kakmakimilh	306T	*Squalus suckleyi*	Not recorded	-11.5	13.4	40.3	14.5	3.2
6445	Kakmakimilh	306T	*Squalus suckleyi*	Vertebrae	-12.6	14.0	39.0	12.9	3.5
6446	Kakmakimilh	306T	*Squalus suckleyi*	Vertebrae	-11.9	14.7	40.0	13.6	3.4

*Specimens with an asterisk indicate collagen samples that were prepped at UBC but measured at the Department of Soil Sciences, University of Saskatchewan.
**Specimens with *UC* indicate collagen samples that were prepped at UBC but measured at the Department of Plant Sciences Stable Isotope Facility, University of California Davis.
***Specimens with *D* indicate duplicate collagen samples for the two dogs recovered from Kakmakimilh (wool dog: 4149 and 4150; small dog: 6538 and 6539).

Table S7. Regional isotope values from domestic dog specimens examined across the Northwest Coast as presented in Figure 3. Values represent averages.

Location	Site	δ^{13}C	SD	δ^{15}N	SD	*n*	Reference
Keatley Creek	EeRI-7	-16.3	0.9	13.8	1.7	6	Barta 2006 [15]
Bridge River	EeRl-4	-15.4	0.4	14.1	0.5	9	Tifental 2015 [16]
Sumas	DgRm-1	-14.8		15.2		1	Barta 2006 [15]
Cathlapotle	45CL1	-14.5	0.6	16.4	0.8	5	Ames et al. 2015 [17]
Namu	EISx-1	-13.0	0.6	16.4	0.8	14	Cannon et al. 1999 [18]
Sliammon	EaSe-2 & DlSd-6	-12.8	0.2	16.3	0.4	2	Zimmerman 2014 [19]
Dionisio	DgRv-3	-12.7	0.4	15.6	0.5	5	Grier 2006 [20]
Gwaii Hannas	785T	-12.2		17.9		1	Szpak et al. 2009 [21]
Port Hardy	EeSu-5	-11.3		16.1		1	Barta 2006 [15]

*

References Cited

1 Schoeninger, M. J. & DeNiro, M. J. Nitrogen and Carbon Isotopic Composition of Bone Collagen from Marine and Terrestrial Animals. *Geochimica et Cosmochimica Acta* **48**, 625–639 (1984).

2 DeNiro, M. J. Postmortem preservation and alteration of in vivo bone collagen isotope ratios in relation to palaeodietary reconstruction. *Nature* **317**, 806–809 (1985).

3 van Klinken, G. Bone Collagen Quality Indicators for Palaeodietary and Radiocarbon Measurements. *J. Archaeol. Sci.* **26**, 687–695 (1999).

4 Szpak, P., Metcalfe, J. Z. & Macdonald, R. A. Best practices for calibrating and reporting stable isotope measurements in archaeology. *J. Archaeol. Sci. Rep.* **13**, 609–616 (2017).

5 Smith, N. F., McKechnie, I., St. Claire, D. E. & Sellers, I. Hup'kisak̲uu7a (93T) – 2015 Archaeological Field Program. (Report submitted to Pacific Rim National Park Reserve, Tseshaht First Nation, and Bamfield Marine Sciences Centre, 2016).

6 Kieser, W. E. Radiocarbon analysis results, UVic 2017 Broken Group Islands Program. (A. E. Lalonde AMS Laboratory Report submitted to the University of Victoria, January 29, 2018).

7 Kieser, W. E. Radiocarbon analysis results, UVic 2018 and 2019 Broken Group Islands Program. (A. E. Lalonde AMS Laboratory Report submitted to the University of Victoria, March, 2020).

8 MacLean, K. *An Analysis of the Flaked Stone Assemblage from the Hiikwis Site Complex, Barkley Sound, British Columbia* MA Thesis, University of Victoria, (2012).

9 Reimer, P. *et al.* The IntCal20 Northern Hemisphere radiocarbon age calibration curve (0-55 kcal BP). *Radiocarbon*, doi:10.1017/RDC.2020.41 (2020).

10 Bronk Ramsey, C. Bayesian Analysis of Radiocarbon Dates. *Radiocarbon* **51**, 337–360 (2009).

11 Alter, S. E., Newsome, S. D. & Palumbi, S. R. Pre-Whaling Genetic Diversity and Population Ecology in Eastern Pacific Gray Whales: Insights from Ancient DNA and Stable Isotopes. *PLoS One* **7**, e35039 (2012).

12 Newsome, S. D. *et al.* The Shifting Baseline of Northern Fur Seal Ecology in the Northeast Pacific Ocean. *PNAS* **104**, 9709–9714 (2007).

13 Markel, R. W. *Rockfish Recruitment and Trophic Dynamics on the West Coast of Vancouver Island: Fishing, Ocean Climate, and Sea Otters* PhD Dissertation, University of British Columbia, (2011).

14 Markel, R. W. & Shurin, J. B. Indirect effects of sea otters on rockfish (*Sebastes* spp.) in giant kelp forests. *Ecology* **96**, 2877–2890, doi:DOI:10.1890/14-0492.1 (2015).

15 Barta, J. L. *Addressing Issues of Domestication and Cultural Continuity on the Northwest Coast Using Ancient DNA and Dogs* PhD Dissertation, McMaster University, (2006).

16 Tifental, E. *The Bridge River Dogs: Interpreting aDNA and Stable Isotope Analysis Collected From Dog Remains* MA Thesis, University of Montana, (2016).

17 Ames, K. M. *et al.* Stable isotope and ancient DNA analysis of dog remains from Cathlapotle (45CL1), a contact-era site on the Lower Columbia River. *J. Archaeol. Sci.* **57**, 268–282 (2015).

18 Cannon, A., Schwarcz, H. P. & Knyf, M. Marine-based subsistence trends and the stable isotope analysis of dog bones from Namu, British Columbia. *J. Archaeol. Sci.* **26**, 399–407 (1999).

19 Zimmerman, K. *Changing Ways, Constant Companions: The Ancient DNA and Local Knowledge of Tla'amin Dogs* MA Thesis, Simon Fraser University, (2014).

20 Grier, C. Affluence on the Prehistoric Northwest Coast of North America In: *Beyond 'Affluent-Foragers': Rethinking Hunter-Gatherer Complexity. Proceedings of the 9th ICAZ Conference, Durham* (eds Colin Grier, Jangsuk Kim, & Junzo Uchiyama) 126–135 (Oxbow Books, 2006).

21 Szpak, P., Orchard, T. J. & Gröcke, D. R. A Late Holocene Vertebrate Food Web from southern Haida Gwaii (Queen Charlotte Islands, British Columbia). *J. Archaeol. Sci.* **36**, 2734–2741 (2009).
